# The Corrosion Behavior of X100 Pipeline Steel in a Sodium Chloride Solution Containing Magnesium and Calcium

**DOI:** 10.3390/ma16155258

**Published:** 2023-07-26

**Authors:** Xiaoning Yang, Tiancong Hao, Qingya Sun, Zhongwei Zhang, Yuan Lin

**Affiliations:** 1 School of Mechanical Engineering, Nanjing University of Science & Technology, Nanjing 210094, China; xiaoninghht@163.com (X.Y.); haotiancong@njust.edu.cn (T.H.); 2 State Key Laboratory of Explosion & Impact and Disaster Prevention & Mitigation, Army Engineering University of PLA, Nanjing 210007, China; zhangzhongwei.cn@gmail.com (Z.Z.); optimus103@mail.ecust.edu.cn (Y.L.)

**Keywords:** X100 pipeline, corrosion scale, calcium, magnesium

## Abstract

The influences of Mg^2+^ and Ca^2+^ on the short-term (1800 s) corrosion behavior of X100 pipeline steel were investigated in a sodium chloride (NaCl) solution saturated with CO_2_. Either Ca^2+^ or Mg^2+^ in the solution inhibited the short-term corrosion of X100 pipeline steel, with the corrosion current density decreasing from 262.4 μA cm^−2^ to 163.5 μA cm^−2^ or 80.8 μA cm^−2^. During longer-term (8−48 h) immersion, the Mg^2+^ inhibited the formation of the protective scale, whereas the Ca^2+^ accelerated the formation of the scale. Further, an experimental equation establishing the relationship between the precipitation rate of the corrosion scale and the exposure time was proposed to quantitatively study the effects of Mg^2+^ and Ca^2+^ on the precipitation rate of the corrosion scale.

## 1. Introduction

The global oil demand will grow from 84 million barrels per day in 2010 to 123 million barrels per day in 2025, as reported by the United States Energy Information Administration [1]. Pipelines are widely used to transport significant amounts of oil [2]. CO_2_ corrosion is a significant problem for pipeline steel. The results of pipeline leakage induced by corrosion are severe [3]. The pipeline operators use some predictive models of CO_2_ corrosion to determine operating parameters, for instance, the pressures, appropriate inhibitors, and flowing velocities [4]. One of the important factors that these prediction models take into consideration is the chemistry of CO_2_-containing water, which may further carry some salts such as magnesium chloride (MgCl_2_) and calcium chloride (CaCl_2_) [4]. The precise understanding of the role of Mg^2+^ and Ca^2+^ in the corrosion of pipeline steel in CO_2_-containing water can help improve these predictive models, which have huge commercial and environmental importance.

The precipitation of protective iron carbonate (FeCO_3_) on steels in CO_2_-containing water needs an induction time [5]. Prior to FeCO_3_ precipitation, general corrosion often occurs in the active zones of steels, such as ferrite [6]. As the concentration of ferrous (Fe^2+^) increases, FeCO_3_ accumulates on the specimen [7]. If Mg^2+^ and Ca^2+^ can affect the initial corrosion prior to FeCO_3_ formation, the addition of Mg^2+^ and Ca^2+^ may influence the subsequent scale precipitation by influencing the initial steel dissolution, which provides Fe^2+^ for the scale formation. Therefore, such additions may be either beneficial (accelerating the formation of the scale) or otherwise (inhibiting the production of the scale) as far as the corrosion rate is considered. However, previous studies [5,7,8,9,10,11,12] only paid attention to the influences of cations on the corrosion of steel after scale precipitation without considering the initial corrosion prior to scale formation.

Further, the role of Mg^2+^ and Ca^2+^ in the corrosion of pipeline steels after the scale formation is under dispute. Jiang et al. stated that the initiation period for localized corrosion on N80 steel in the NaCl solutions containing 1.5 wt.% CaCl_2_ is prolonged and the corrosion rate is inhibited by the presence of Ca^2+^ at 57 °C [11]. Pots reported that a more porous and less protective scale that consists of iron and calcium carbonate can precipitate in the CO_2_ solution with the addition of Ca^2+^ [12]. However, Esmaeely et al. showed that low Ca^2+^ concentrations (10 ppm and 100 ppm) in solutions decrease the corrosion rates of steel at 80 °C because of the production of FeCO_3_ [7]. When the Ca^2+^ concentration exceeds 1000 ppm, a non-protective calcium carbonate (CaCO_3_) scale forms and the corrosion of steel deteriorates [7]. With regard to magnesium chloride (MgCl_2_), it has been proposed that MgCl_2_ additions decrease the required critical supersaturation for FeCO_3_ precipitation in the case of carbon steel immersed in the NaCl solutions [5]. In contrast, Chen et al. argued that the additions of Mg^2+^ restrain the formation of scales, both in the bulk solutions as well as on the stainless steel [13]. Additionally, during the establishment of various prediction models of CO_2_ corrosion, the precipitation rate of the corrosion scale needs to be considered and calculated [14,15]. However, to the best of our knowledge, there is no research study concerning the quantitative effects of Ca^2+^ and Mg^2+^ on the formation rate of the corrosion scale, which is an important parameter for establishing the prediction models.

In this study, the influences of Mg^2+^ and Ca^2+^ on the short-term (prior to scale formation) corrosion behavior of X100 pipeline steel were investigated by open circuit potential (OCP), potentiodynamic polarization (PDP), and scanning electron microscopy (SEM). Further, OCP, linear polarization resistance (LPR), and electrochemical impedance spectroscopy (EIS) were employed to study the longer-term (after scale formation) corrosion behavior of X100 pipeline steel. SEM, X-ray diffraction (XRD), and X-ray photoelectron spectroscopy (XPS) were utilized to characterize the surface information of corrosion scales. Especially, the quantitative effects of Ca^2+^ and Mg^2+^ on the formation rate of the corrosion scale were gained. The results will help improve various predictive models that are widely used to evaluate the CO_2_ corrosion of steel pipelines.

## 2. Experimental

### 2.1. Materials and Electrolytes

The composition of the X100 pipeline steel in this study is given in Table 1. The specimens for electrochemical measurements were connected to a copper wire. The specimens sealed by the epoxy had a 1 cm^2^ front face. The specimens were sequentially ground with SiC papers of 120, 600, 800, and 1200 grit.

Deionized (DI) water, NaCl (Anachemia), anhydrous MgCl_2_ (Amresco), anhydrous CaCl_2_ (Anachemia), and sodium hydroxide (NaOH, Anachemia) were used to prepare the solutions. The desired Mg^2+^ and Ca^2+^ concentrations were achieved by the addition of MgCl_2_ and CaCl_2_. Table 2 presents the chemical compositions of the solutions in this work. The addition of CaCl_2_ and MgCl_2_ introduces extra Cl^−^ ions that may influence the corrosion rate of steel; thus, all the solutions listed in Table 2 have the same Cl^−^ concentration, which was adjusted with the NaCl concentration. The solutions were sparged with a 1-bar CO_2_ for 2 h before all the measurements were taken. Then, the specimens were placed in the solutions, and CO_2_ sparging was continued throughout the measurements. The solution temperature was maintained at 60 ± 1 °C. The pH values of the solutions were controlled to 7 ± 0.2 by the addition of NaOH. The pH values were tested by an ALpHA^Ⓗ^ series epoxy electrode manufactured by OMEGA.

### 2.2. Electrochemical and Surface Analysis Measurements

A Princeton Applied Research Versastat 4 was utilized for the various electrochemical tests. The standard three-electrode system for electrochemical measurements used graphite as the counter electrode, a saturated calomel electrode (SCE, 0.215 V vs. SHE) as the reference electrode, and X100 as the working electrode. The short-term OCP measurements were conducted for 1800 s. The longer-term corrosion behavior of the specimens was measured by OCP with 1 point per hour. At every 8 h interval within the OCP measurements, EIS and LPR measurements were conducted to characterize the scale evolution. LPR measurements were carried out from −0.02 to 0.02 V (vs. OCP) with a scan rate of 0.167 mV s^−1^. EIS measurements were tested in the frequency range of 0.01 Hz−10 kHz with a sampling rate of 10 points per decade. EIS data were analyzed and fitted by ZSimpWin. Here, it is assumed that EIS and LPR measurements would not influence the real evolution of longer-term OCP since they do not significantly polarize the samples during the measurements [16]. All the electrochemical measurements were repeated at least three times in order to obtain reliable results.

Surface corrosion morphology of the specimens was characterized by a field-emission scanning electron microscope (SEM, Zeiss Σigma, Shenzhen, China). X100 pipeline steel with corrosion scales on the surface was carbon-coated to mitigate the charging effects. The SEM measurements were repeated at three different positions in the selected area to ensure reliable results. The crystal structures of the corrosion scales were identified by a Rigaku MultiFlex machine (manufactured by Hangzhou Remai Technology Co., Ltd., Hangzhou, China) using Cu Kα radiation (wavelength: 0.15406 nm). All XRD measurements were completed from 20 deg to 55 deg with a rate of 0.5 deg min^−1^. The XRD measurements were repeated three times to ensure consistent results. XPS was tested on an Omicron & Leybold MAX200 with a monochromated Al Kα source (1486.6 eV). The absolute binding energies were calibrated by the C 1s line of adventitious carbon at 284.8 eV binding energy. Wide spectra were collected from areas of 300 μm × 300 μm, at ~10 nm depth, using a pass energy of 100 eV. High-resolution spectra were collected at the binding energies of Fe, O, C, Ca, Mg, and Mn using a pass energy of 20 eV. Shirley and linear backgrounds were used in the curve fitting process performed in XPS Peak software (XPS Peak Fit V4.1).

## 3. Results and Discussion

### 3.1. Effects of Mg^2+^ and Ca^2+^ on the Short-Term Corrosion Behavior of X100

**Figure 1:**
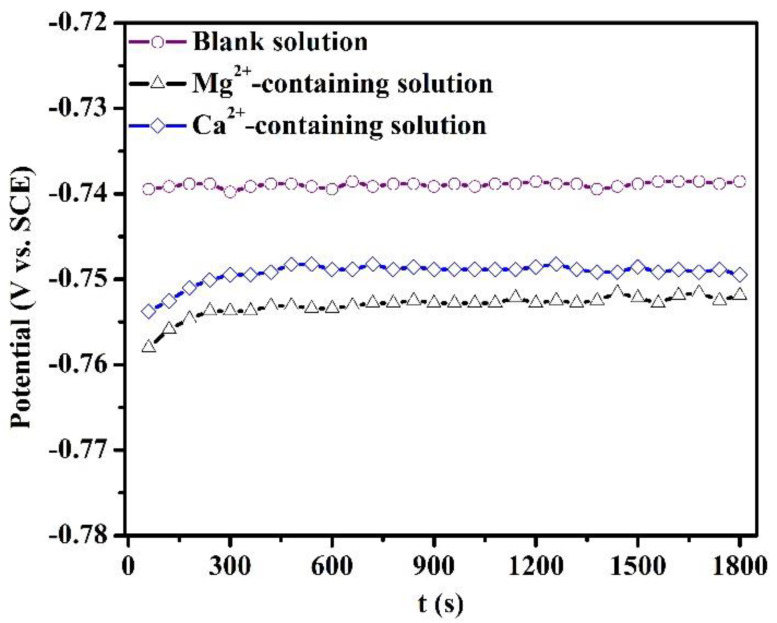
Open circuit potential of the specimens after 1800 s of immersion in the blank solutions with various additions.

Figure 1 depicts the variation of OCP of specimens after 1800 s of immersion in the blank solutions with various additions. The fluctuation of OCP over the last 10 min of each OCP measurement in Figure 1 is less than 5 mV. The addition of Mg^2+^ or Ca^2+^ causes a shift of OCP to the cathodic direction. The maximum shift in the OCP was 15 mV for the Mg^2+^-containing solution. The presence of Ca^2+^ and Mg^2+^ can inhibit the cathodic reaction, thus decreasing the OCP.

**Figure 2:**
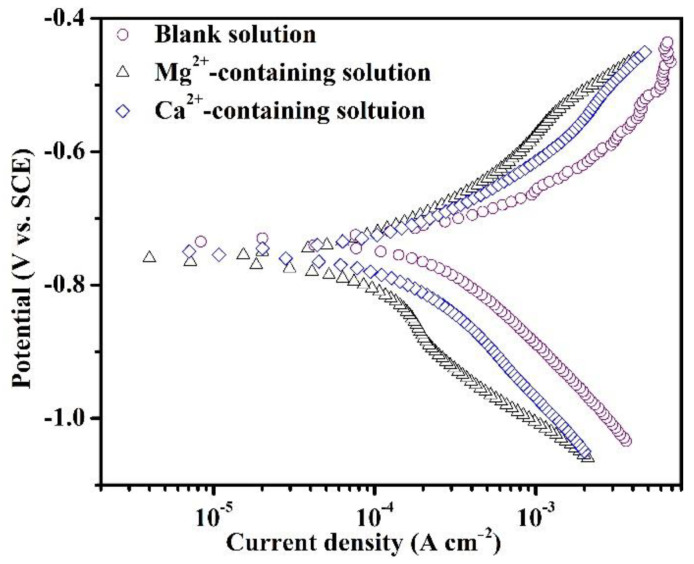
Potentiodynamic polarization plots of the specimens after 1800 s of immersion in the blank solutions with various additions.

Figure 2 shows the PDP plots of the specimens in the blank solutions with various additions after 1800 s of immersion. The short immersion was selected prior to the precipitation of large amounts of corrosion products, thus enabling the study of the effects of different cations on the electrochemical reactions in the initial stage of corrosion. As reported in the literature [17], the cathodic process (1, 2, and 3) and dissolution behavior (4) should have proceeded in the blank solutions, depending on the following reactions:

(1) $$2H^{+}+2e^{-}=H_{2}\;$$

(2) $$2H{\mathrm{CO}}_{3}^{-}+2e^{-}=2{\mathrm{CO}}_{3}^{2-}+H_{2}\;$$

(3) $$2H_{2}O+2e^{-}=H_{2}+OH^{-}\;$$

(4) $${\mathrm{Fe}=\mathrm{Fe}}^{2+}+2e^{-}\;$$

The related electrochemical parameters of PDP plots in Figure 2 are shown in Table 3. The presence of Mg^2+^ and Ca^2+^ decreases the *i_corr_* values, illustrating the inhibiting role of Mg^2+^ and Ca^2+^ in the corrosion of specimens. According to the *i_corr_* values, Mg^2+^ presents a better impeding effect compared to Ca^2+^. The negative shift of *E_corr_* values from −733.7 mV vs. SCE to −756.7 mV vs. SCE and −751.3 mV vs. SCE indicates an inhibition effect of cations on the cathodic reaction. The decrease in *i_corr_* after adding cations indicates a sluggish kinetics effect of cations during the initial corrosion process. The *b_c_* values for all three samples are more than 140 mV dec^−1^, indicating that the Volmer step is the rate-determining step of various processes involved in cathodic reactions.

**Figure 3:**
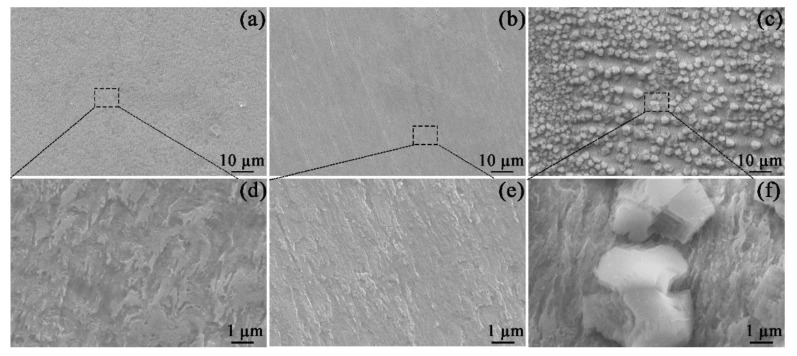
SEM images of the corrosion morphology on the specimens exposed to the blank solutions with no addition (**a**,**d**), Mg^2+^ addition (**b**,**e**), and Ca^2+^ addition (**c**,**f**) for 1800 s.

As shown in Figure 3, the SEM images of specimens were measured at different magnifications. After 1800 s of exposure, the corrosion scale did not form (Figure 3a,b). In Figure 3a,b, the specimen surface was highly corroded by virtue of the fast dissolution of the steel. When Mg^2+^ was added to the solution (Figure 3b,e), the steel surface after 1800 s of immersion still had scratches from the sample preparation, implying a relatively slow corrosion rate. The comparison of Figure 3d,e confirms that the presence of Mg^2+^ can inhibit the corrosion of the specimen. Interestingly, when Ca^2+^ was added to the solution (Figure 3c), large amounts of particles appeared on the steel surface. Per the Eh−pH diagram of Ca−C−H_2_O (see the supplementary information), at 60 °C and pH 7, the stable CaCO_3_ has formed in Ca^2+^-containing solution. However, no particle was seen in the Mg^2+^-containing solution (Figure 3b), owing to the higher solubility of MgCO_3_ compared to that of CaCO_3_ (see Eh−pH diagram of Mg−C−H_2_O in the supplementary information).

As stated in the introduction, the effects of Mg^2+^ and Ca^2+^ on the CO_2_ corrosion of steel prior to scale formation have previously been unobtainable. Therefore, this part mainly investigated the short-term corrosion evolution of X100 steel in the blank solutions with various additions by OCP, PDP, and SEM measurements. After 1800 s of exposure, the corrosion scale was not detected on the specimens. Either Ca^2+^ or Mg^2+^ in solutions can restrain the corrosion of X100 pipeline steels when the steels are placed in these solutions for 1800 s. Collazo et al. [18] found a similar result that Mg^2+^ in chloride-containing electrolytes (without purging CO_2_) can act as a corrosion inhibitor for aluminum alloy samples by inhibiting the cathodic reactions. In addition, some metal cations were used as corrosion inhibitors for mild steel in a sulfuric acid solution [19]. Actually, the OCP shift (Figure 1) for the solutions containing Mg^2+^ and Ca^2+^ demonstrates that these cations can inhibit the corrosion rate by suppressing the cathodic reactions.

### 3.2. Effects of Mg^2+^ and Ca^2+^ on the Compositions of Corrosion Scale

**Figure 4:**
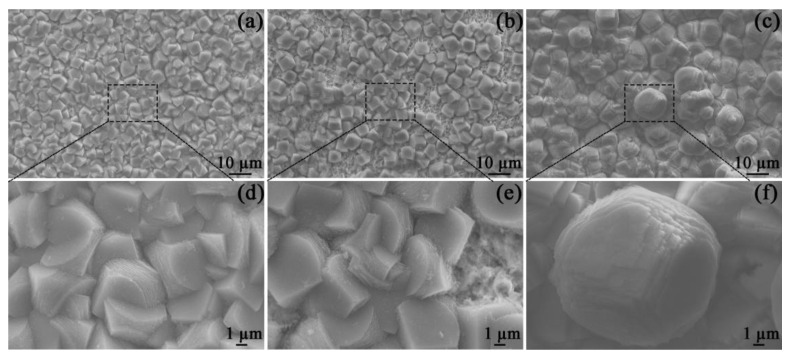
SEM images of the corrosion scales on the specimens placed in the blank solutions with no addition (**a**,**d**), Mg^2+^ addition (**b**,**e**), and Ca^2+^ addition (**c**,**f**) for 48 h.

After 48 h of OCP measurements, the corrosion scales of the specimens in all solutions were observed by SEM (Figure 4). All specimen surfaces were covered with porous and prismatic crystals, which are characterized by irregular edges and stepped growth morphology. These characteristics are in line with the morphologies of FeCO_3_ scales that are frequently detected in similar environments [20].

**Figure 5:**
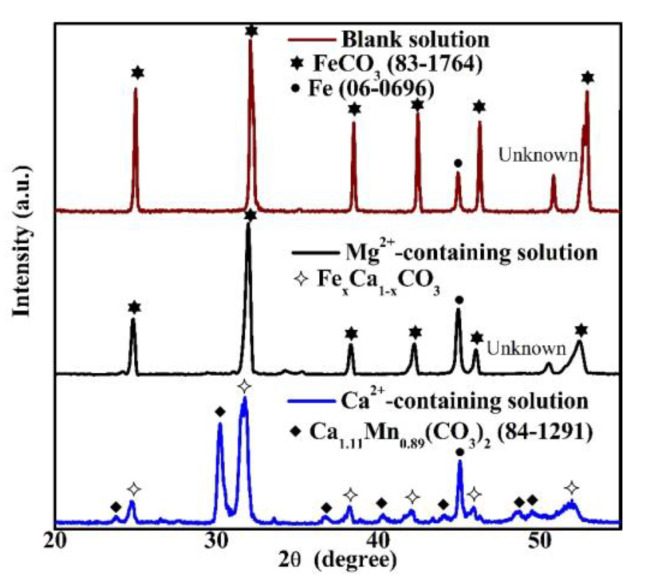
The XRD pattern of the corrosion scales formed on the specimens exposed to the blank solutions with various additions for 48 h.

XRD measurements were conducted in order to analyze the crystal structures in Figure 4. When the specimen was put in the blank solution, the notable crystal structure in the corrosion scale was FeCO_3_, as shown in Figure 5. This crystal structure is determined based on the database of the International Centre for Diffraction Data [21]. For the corrosion scale in the solution containing Mg^2+^, only FeCO_3_ is recognized (Figure 5). However, if Ca^2+^ is present, the corrosion scale consists of Fe_x_Ca_1−x_CO_3_ and Ca_1_._11_Mn_0_._89_(CO_3_)_2_ (84–1291) [21]. The identification of Fe_x_Ca_1−x_CO_3_, which is an intermediate compound between CaCO_3_ and FeCO_3_, is obtained based on the XRD results of Esmaeely et al. [8]. The Ca_1_._11_Mn_0_._89_(CO_3_)_2_ identified in the corrosion scale may be related to the dissolution of MnS in X100 pipeline steel [22,23].

**Figure 6:**
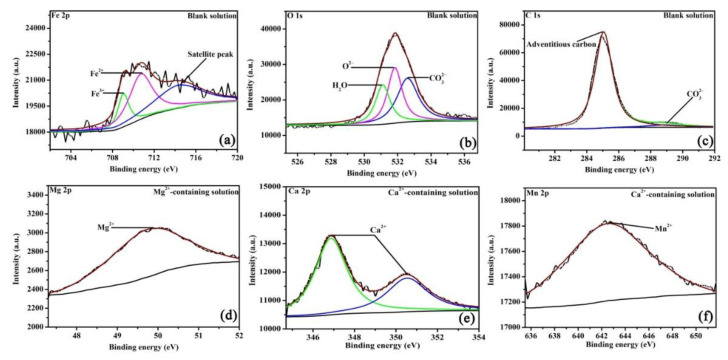
The XPS pattern of the corrosion scales of the specimens exposed to the blank solutions with no addition (**a**–**c**), Mg^2+^ addition (**d**), and Ca^2+^ addition (**e**,**f**) for 48 h.

As XRD cannot detect the amorphous phase or trace amounts of crystalline structures, XPS was tested to precisely analyze the valence states of elements in the corrosion scales. Figure 6 shows the high-resolution XPS pattern of corrosion scales on specimens after 48 h of OCP measurements. Fe, O, and C elements are present on all scales and formed in different solutions. In the blank solution (Figure 6a), the Fe 2p peaks at 709.9, 710.9, and 714.5 eV, which are assigned to Fe^3+^, Fe^2+^, and the satellite peak, respectively [24,25]. In Figure 6b, the O 1s peaks at 530.9, 531.7, and 532.7 eV, which are ascribed to the presence of H_2_O, O^2−^, and CO_3_^2−^, consecutively [24,25]. Fe^3+^ and O^2−^ correspond to Fe_2_O_3_, while Fe^2+^ and CO_3_^2−^ are attributed to FeCO_3_. However, it is unclear whether Fe_2_O_3_ formed in the solution or resulted from oxidation after being removed from the solution [22]. Figure 6c shows a small peak at 289.6 eV, which is attributed to CO_3_^2−^. For the Mg^2+^-containing solution, apart from the peaks assigned to Fe_2_O_3_ and FeCO_3_ (see supplementary information), the peak of Mg^2+^ (Figure 6d) appears at 49.9 eV, which belongs to MgCO_3_. MgCO_3_ may be produced by the local pH increase as a result of cathodic reactions (2) and (3). XRD cannot find MgCO_3_ in the corrosion scale, either because MgCO_3_ is amorphous or because the content of MgCO_3_ (even if it is crystalized) is low. When Ca^2+^ is added (Figure 6e), the fitting peaks for Ca 2p appear at 346.8 and 350.5 eV, which can reflect Fe_x_Ca_1−x_CO_3_ in the corrosion products [26]. In Figure 6f, the peaks of Mn are seen in the scale in Ca^2+^-containing solution, which further identifies the formation of Ca_1_._11_Mn_0_._89_(CO_3_)_2_ [26]. The XPS plots demonstrate that either Mg^2+^ or Ca^2+^ in the NaCl solution saturated with CO_2_ can affect the scale structures by forming carbonate species. The concentration of various bonds identified in the current study is important for determining the corrosion process of steel; this information will be provided in a future study.

In the literature, it is widely accepted that Ca^2+^ can be solutionized within the FeCO_3_ crystal, and Fe_x_Ca_1−x_CO_3_ formed in the solutions containing CO_2_ and Ca^2+^ [8,9,27]. As for Mg^2+^, Yu et al. [9] thought that carbonate species containing Mg^2+^ can form in the solutions containing CO_2_ and Mg^2+^, whereas Ingham et al. [5] used in situ XRD to measure the composition of the corrosion scale and reported that the Mg^2+^ in CO_2_-containing solutions cannot lead to the formation of detectable Mg^2+^-containing carbonate species. Therefore, both XRD and XPS were used to precisely measure the compositions of the corrosion scale to solve the current dispute about whether Mg^2+^-containing carbonate species form in the corrosion scale. This study reveals that either amorphous MgCO, trace amounts of crystalline MgCO_3_, or both exist in the corrosion scale.

### 3.3. Effects of Mg^2+^ and Ca^2+^ on the Longer-Term Corrosion Behavior of X100

**Figure 7:**
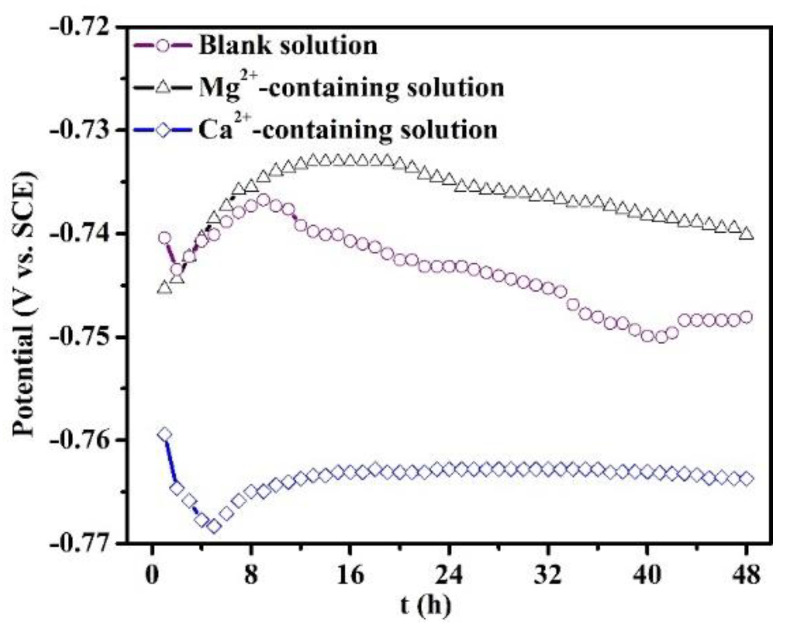
Open circuit potential evolution of the specimens put in the blank solutions with various additions for 48 h.

The evolution of OCP of the specimens in all the solutions for 48 h is recorded in Figure 7. The OCP curve of the specimen in the blank solution is characterized by an initial increase and reaches a maximum value at 8 h, and then drops after 8 h. When the immersion time is more than 40 h, the curve displays a stable fluctuation. The presence of Ca^2+^ in the solution decreases the OCP value with respect to the blank solution, and the curve presents a stable fluctuation only after 16 h of immersion. This demonstrates that the Ca^2+^ addition predominantly suppresses the cathodic reactions. However, the existence of Mg^2+^ causes a positive OCP shift, with the corrosion potential reaching a maximum value at 16 h.

**Figure 8:**
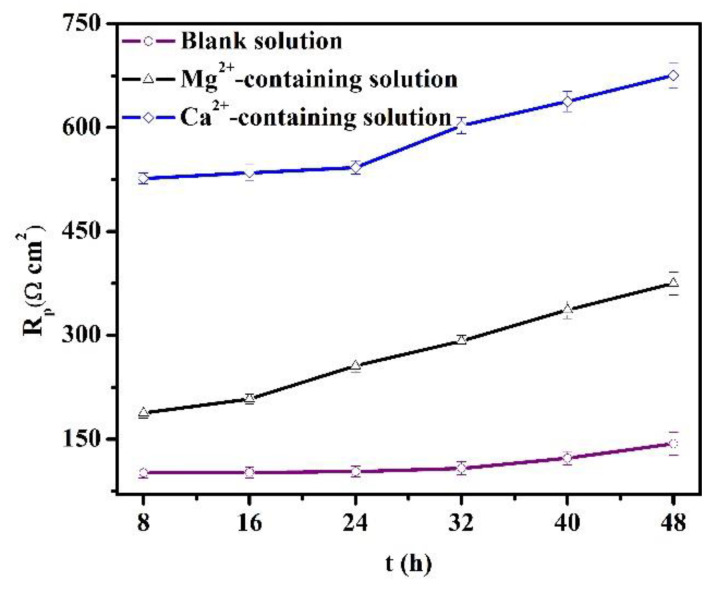
Polarization resistances obtained from the linear polarization method for the specimens in the blank solutions with various additions.

During the OCP tests, LPR was also measured to understand the evolution of longer-term corrosion. In Figure 8, the *R_p_* values increase versus time for the specimens in all the solutions. The increase in the *R_p_* values is usually attributed to the thickening of the protective scale [28]. Also, the Ca^2+^ addition significantly amplifies the *R_p_* values, while the Mg^2+^ in the solution causes a negative shift of the *R_p_* values. As indicated in Section 3.1, the Mg^2+^ in the solution can inhibit the short-term corrosion of specimens. Thus, the concentration of Fe^2+^ produced by the steel dissolution declines, and the precipitation rate of protective scale is impeded. Lower *R_p_* values for specimens in the Mg^2+^-containing solution were triggered by the relatively low formation rate of the corrosion scale. In contrast, the presence of Ca^2+^ in the solution accelerated the formation of corrosion scale.

**Figure 9:**
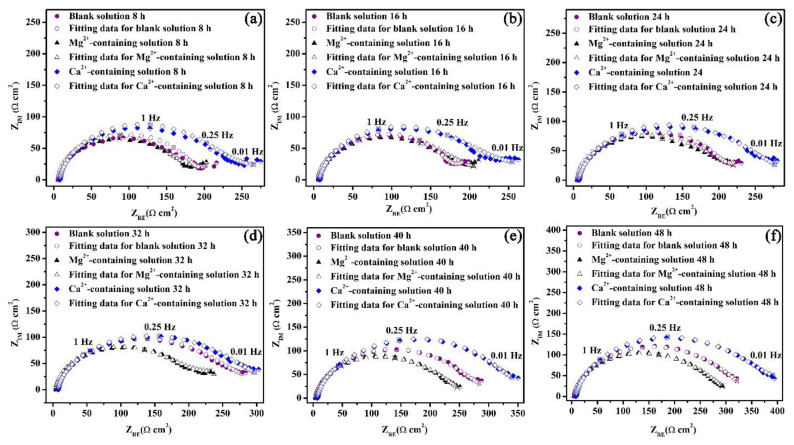
Nyquist plots for the specimens in the blank solutions with various additions for 8 (**a**), 16 (**b**), 24 (**c**), 32 (**d**), 40 (**e**), and 48 (**f**) h.

**Figure 10:**
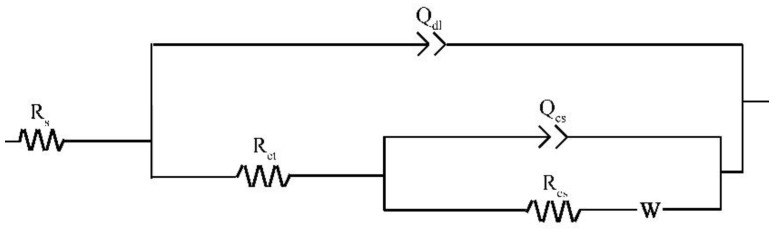
Equivalent circuit model for electrochemical impedance spectroscopy.

The Nyquist plots for the specimens exposed to the three solutions for 48 h are shown in Figure 9. To make a qualitative comparison of the curves presented in Figure 9, it is necessary to model the datasets with electrical equivalent circuits. *R_s_*(*Q_dl_*(*R_ct_*(*Q_cs_*(*R_cs_W*)))) is used to interpret the steel/solution interface of this study (Figure 10) with the Warburg element selected based on a previous study [29]. The circuit elements and their notations in Figure 10 are as follows: *R_s_* is the solution resistance, *Q_dl_* is the constant phase element (CPE) for the electrical double layer, *R_ct_* is the charge transfer resistance, *R_cs_* is the corrosion scale resistance, *Q_cs_* is the CPE for the corrosion scale, and W is the Warburg diffusion element. The chosen equivalent circuit gives good fits between the simulated and measured results for all cases in this work. The values of Chi-Square (*X*^2^) for all the EIS data are approximate 1 × 10^−4^ (Supplementary information).

**Figure 11:**
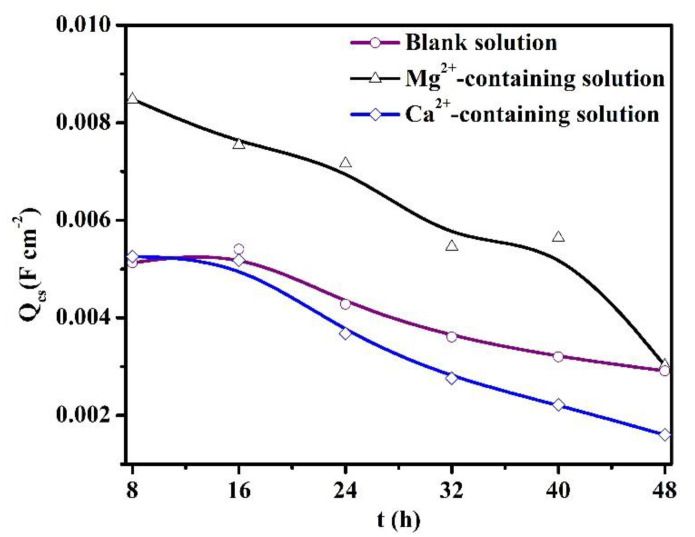
The values of *Q_cs_* for the specimens exposed to the blank solutions with different additions.

The *Q_cs_* values are presented in Figure 11. The *Q_cs_* reflects the available area for the cathodic reaction, which predominantly occurs at the cementite of carbon steel [30]. For the blank solutions, the *Q_cs_* values decline as time increases, illustrating the thickening of the FeCO_3_ scales and the decrement of the available cathodic area [30]. Thus, the cathodic reactions are predominantly suppressed, which is corroborated by the decline in OCP values after 8 h of immersion (Figure 7). The variations of *Q_cs_* values, *Rp* (Figure 8), and the thickness of the corrosion scale (see the supplementary information) validate the thickening of the protective corrosion scale over time. In addition, when the X100 pipeline steels were placed in three solutions for the same amount of time, the presence of Ca^2+^ decreased the *Q_cs_* values, while the addition of Mg^2+^ increased the *Q_cs_* values. One explanation for the variations of *Q_cs_* values would be the different formation rates of corrosion scales when Ca^2+^ or Mg^2+^ are present. The Mg^2+^-containing solution is characterized by the relatively low formation rate of the protective scale, while the presence of Ca^2+^ in a solution can accelerate the formation of the protective scale.

Figure 8 and Figure 11 demonstrate that the thickening of the protective corrosion scale is beneficial for corrosion reduction in pipeline steels. Traditionally, it is thought that the coexistence of Mg^2+^ and Ca^2+^ in CO_2_-containing solutions can produce heavy carbonate precipitation, which may be (Fe, Ca, Mg)CO_3_ [9,31,32]. However, that does not indicate that Mg^2+^ alone in the solution can accelerate the formation of the scale. This study reveals that Mg^2+^ alone in the solution inhibits the scale precipitation, while the Ca^2+^ in the solution indeed accelerates the formation of the protective scale.

Although some studies on the corrosion scale have been carried out in the NaCl solutions containing CO_2_, these studies focused on the evolutions in the scale composition and corrosion rate by altering the pH [33], temperature [34], CO_2_ partial pressure [35], and chloride concentrations [36]. Further, some models were proposed to establish the relationship between the precipitation rate of the CO_2_ scale and the supersaturation of the solution [37,38]. For instance, van Hunnik et al. reported the following [37]:

(5) $$S=\frac{C_{{\mathrm{Fe}}^{2+}}C_{{\mathrm{CO}}_{3}^{2-}}}{K_{SP}}$$

where *S* is the supersaturation, *K_SP_* is the solubility product of FeCO_3_, and *C* represents the concentration. Then, the precipitation rate of FeCO_3_ follows the equation [37]:

(6) $${\left[{\mathrm{Fe}}^{2+}\right]}_{prec}=k_{r}\frac{A}{V}K_{SP}(S-1)(1-S^{-1})exp\left(52.4-\frac{119.8}{RT}\right)$$

where [Fe^2+^]*_prec_* is the precipitation rate of FeCO_3_, *A*/*V* corresponds to the surface area-to-volume ratio, *k_r_* stands for the kinetic constant, *K_SP_* represents the solubility product of FeCO_3_, *R* is the universal gas constant, and *T* corresponds to the temperature. However, during corrosion development, the concentration of Fe^2+^ is not constant with time. On the one hand, the formation of the corrosion scale consumes Fe^2+^. On the other hand, the dissolved steel is weakened due to the improved protection of the corrosion scale with time. Therefore, the precipitation rate of the corrosion scale may change with the variance of the Fe^2+^ concentration. It is inconvenient to constantly measure the Fe^2+^ concentration in pipelines, especially when the pipelines are running. Liu et al. [36] investigated the effects of the chloride content on the CO_2_ corrosion of carbon steel based on the point defect model (PDM), which considers the migration of oxygen vacancy and metal vacancy during scale growth. According to the PDM, when the thickness is more than 5 Å, the following equation is derived [16]:

(7) $$L=\frac{RT}{2F\varepsilon }\left(ln\left(\frac{2F\varepsilon A\left(B-1\right)}{RT}\right)+ln\left(t\right)\right)$$

where *R* is the universal gas constant, *T* corresponds to the temperature, *ε* represents the electrical field strength, *F* stands for the faradic constant, *A* and *B* are constant, and *t* is the measurement time. Nevertheless, the relationship between scale thickness and time was not checked by Liu et al. to evaluate whether PDM applied to the scale formation in their study. Collectively speaking, these studies are inconvenient for predicting the precipitation rate of the corrosion scale, and they fail to consider the effects of Mg^2+^ and Ca^2+^ on the precipitation rate of the corrosion scale.

**Figure 12:**
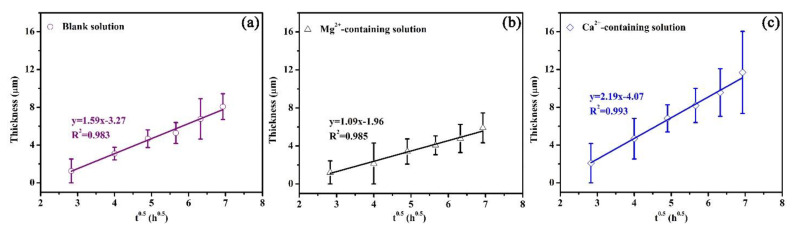
Plots of average scale thickness versus time for the corrosion scales on the specimens exposed to the blank solution (**a**), Mg^2+^-containing solution (**b**), and Ca^2+^-containing solution (**c**).

In this study, an experimental equation was proposed to establish the relationship between the thickness of the corrosion scale and exposure time, which is similar to Equation (7). Based on the experimental equation, the quantitative effects of Mg^2+^ and Ca^2+^ on the precipitation rate can be obtained. Figure 12 shows the plots of the average scale thickness (*L*) versus time (*t*) for the corrosion scales on the specimens exposed to the blank solutions with various additions. The average thicknesses of the corrosion scales were measured by SEM (Figures S4–S6 in the supplementary information). The minimum correlation coefficient (*R^2^*) resulting from *L* versus *t*^0.5^ was 0.983 (Figure 12a), demonstrating that *L* and *t*^0.5^ show a good linear relationship. The scale growth obeys the following relationship:

(8) $$L=At^{0.5}+B$$

(9) $$\frac{dL}{dt}=0.5At^{-0.5}$$

where *L* is the average scale thickness, *A* and *B* are the constant, and *t* is the time. From Figure 12, the values of *A* were calculated. The values of *A* for three solutions were 1.59, 1.09, and 2.19 µm/h^0.5^, respectively. When the specimens were exposed to the solutions for the same time, the presence of Ca^2+^ benefits the growth of the corrosion scale while the Mg^2+^ in the solution weakens the growth rate of the corrosion scale.

According to the above results, it is reasonable to make the following hypothesis on the roles of Ca^2+^ and Mg^2+^ in the formation of a corrosion scale. Fe^2+^ produced by the dissolution of the specimen (reaction (4)) reacts with a carbonate (CO_3_^−^) that is formed due to the reduction in bicarbonate (reaction (2)), which leads to the precipitation of the FeCO_3_ scale on the X100 pipeline steel [28]. The corroded matrix of X100 pipeline steel behaved as a nucleation skeleton, promoting the formation of the scale [28]. Though Mg^2+^ addition can trigger a decrease in the formation rate of the corrosion scale, the sample with Mg^2+^ addition still showed a decrease in the corrosion rate due to the inhibition effect of Mg^2+^ in the electrolyte (Figure 2 and Figure 3). This reduced the available nucleation skeleton and the Fe^2+^ concentration for the growth of the corrosion scales. Correspondingly, the formation rate of the corrosion scale in Mg^2+^-containing solutions was prohibited. It is assumed that the inhibition effect of Mg^2+^ can persist even after the formation of a corrosion scale. But the specimen in the Mg^2+^-containing solution showed the minimum *Rp* values (Figure 8) and maximum *Q_cs_* values due to the low formation rate of the corrosion scale. As for the solution with Ca^2+^, the formation of CaCO_3_ particles on the surface of X100 pipeline steel predominantly benefits the precipitation of the corrosion scale by acting as the nucleation sites of the corrosion scale (Figure 3 and Figure 4). As a result, the Ca^2+^-containing solution presents the thickest scale (Figure 12).

## 4. Conclusions

The role of Mg^2+^ and Ca^2+^ in the corrosion of pipeline steels after scale formation is under dispute in the corrosion field. Thus, the current study was conducted to clarify the discrepancy between different studies and provide a deep understanding of the cation effects in the corrosion process. The following conclusions are drawn based on the results of this work:

1. The corrosion of X100 pipeline steel (*i_corr_* 262.4 μA cm^−2^) after 1800 s of exposure was inhibited by the presence of either Ca^2+^ (*i_corr_* 163.5 μA cm^−2^) or Mg^2+^ (*i_corr_* 80.8 μA cm^−2^) in the NaCl solution saturated with CO_2_.
2. Either Mg^2+^ or Ca^2+^ in the NaCl solution saturated with CO_2_ can affect the scale structures by forming carbonate species. Though Mg^2+^ addition can trigger a decrease in the formation rate of the corrosion scale, the sample with Mg^2+^ addition still shows a decrease in the corrosion rate due to the inhibition effect of Mg^2+^ in the electrolyte.
3. The Ca^2+^ in the solution accelerates the formation of the protective scale in the NaCl solution saturated with CO_2_, thus improving the corrosion resistance of carbon steel.

## Figures and Tables

**Table 1:** The chemical composition of X100 pipeline steel.

Element	C	Mo	Al	Mn	Cu	Ni	Ti	Cr	V	Nb
X100 (wt.%)	0.098	0.20	0.021	1.665	0.25	0.132	0.02	0.017	0.003	0.044

**Table 2:** The chemical compositions of the solutions in this study.

Composition	NaCl (g L^−1^)	CaCl_2_ (g L^−1^)	MgCl_2_ (g L^−1^)	Total Cl^−^ (g L^−1^)
Blank solution	0.7	0	0	0.425
Mg^2+^-containing solution	0.456	0	0.2	0.425
Ca^2+^-containing solution	0.290	0.389	0	0.425

**Table 3:** Polarization parameters for specimens after 1800 s of immersion in the blank solutions with various additions. *E_corr_* is the corrosion potential, *i_corr_* stands for the corrosion current density, and *b_c_* as well as *b_a_* correspond to the Tafel slope.

Solution	*E_corr_* (mV vs. SCE)	*i_corr_* (μA cm^−2^)	*b_c_* (mV dec^−1^)	*b_a_* (mV dec^−1^)
Blank solution	−733.7	262.4	254	155
Mg^2+^-containing solution	−756.7	80.8	187	198
Ca^2+^-containing solution	−751.3	163.5	276	179

## Data Availability

The data that support the findings of this study are available upon request from the corresponding author upon reasonable request.

## Supplementary Materials

The following supporting information can be downloaded at: https://www.mdpi.com/article/10.3390/ma16155258/s1. References [29,39,40,41,42] were cited in supplementary materials.
