# Supplementary material for: The Corrosion Behavior of X100 Pipeline Steel in a Sodium Chloride Solution Containing Magnesium and Calcium
Source: Materials (Basel). 2023 Jul 26;16(15):5258. doi: 10.3390/ma16155258 (PMC10420105; doi:10.3390/ma16155258)
Supplement: Supplementary file 1 [file materials-16-05258-s001.zip › materials-2520342-supplementary.pdf]

materials

Supplementary Information

Effects of Dissolved Magnesium and Calcium on the Corrosion behavior of X100 Pipeline Steel in sodium chloride solution saturated with CO_2

Xiaoning Yang [1,2], Tiancong Hao [1], Qingya Sun [1,*], Zhongwei Zhang [2] and Yuan Lin [2]

1 School of Mechanical Engineering, Nanjing University of Science & Technology, Nanjing 210094, China; xiaoninghht@163.com (X.Y.); haotiancong@njust.edu.cn(T.H.)
2 State Key Laboratory of Explosion & Impact and Disaster Prevention & Mitigation, Army Engineering University of PLA, Nanjing 210007, China; zhangzhongwei.cn@gmail.com (Z.Z.); optimus103@mail.ecust.edu.cn(Y.L.)
* Correspondence: qingyasun@sina.com (Q.S.)

1.1. Effects of Mg^{2+} and Ca^{2+} on the short-term corrosion behavior of X100

It is found in the main text that when the Ca^{2+} was added to the solution (Fig. 3(c)), a large number of particles appeared on the steel surface, whereas no particle was seen in the Mg^{2+}-containing solution (Fig. 3(b)). Per the Eh–pH diagram (Fig. S1), at pH 7, the stable $CaCO_3$ has formed in the Ca^{2+}-containing solution, while the stable $MgCO_3$ cannot form in the Mg^{2+}-containing solution.

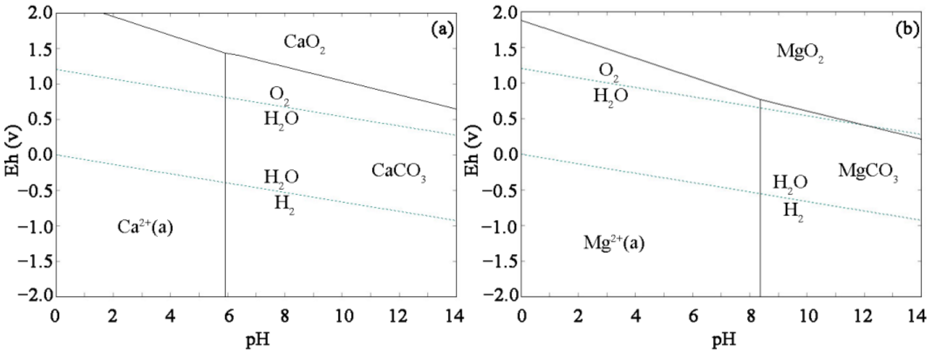

Figure S1. Eh–pH diagrams in the Ca–C–H_2O (a) and Mg–C–H_2O (b) systems with [Ca]= 0.00375 mol L^{-1}, [Mg] = 0.002 mol L^{-1} and [CO_3^{2-}] = 0.027 mol L^{-1} at 333 K. The dashed lines represent the hydrogen evolution and oxygen reduction, respectively. The diagrams were drawn by HSC chemistry software (Outokompou Research, Finland).

1.2. Effects of Mg^{2+} and Ca^{2+} on the compositions of corrosion scale

It is stated in the main text that Fe, O, and C elements are present on all the scales formed in different solutions. Fig. S2 shows the XPS pattern of the corrosion scales on the specimens in the blank solutions with Mg^{2+} addition and Ca^{2+} addition. The peaks of Fe 2p, O 1s, and C 1s can be detected on the scales formed in the two solutions. The Fe^{3+} and O^{2-} correspond to Fe_2O_3, and Fe^{2+} and CO_3^{2-} are attributed to $FeCO_3$.

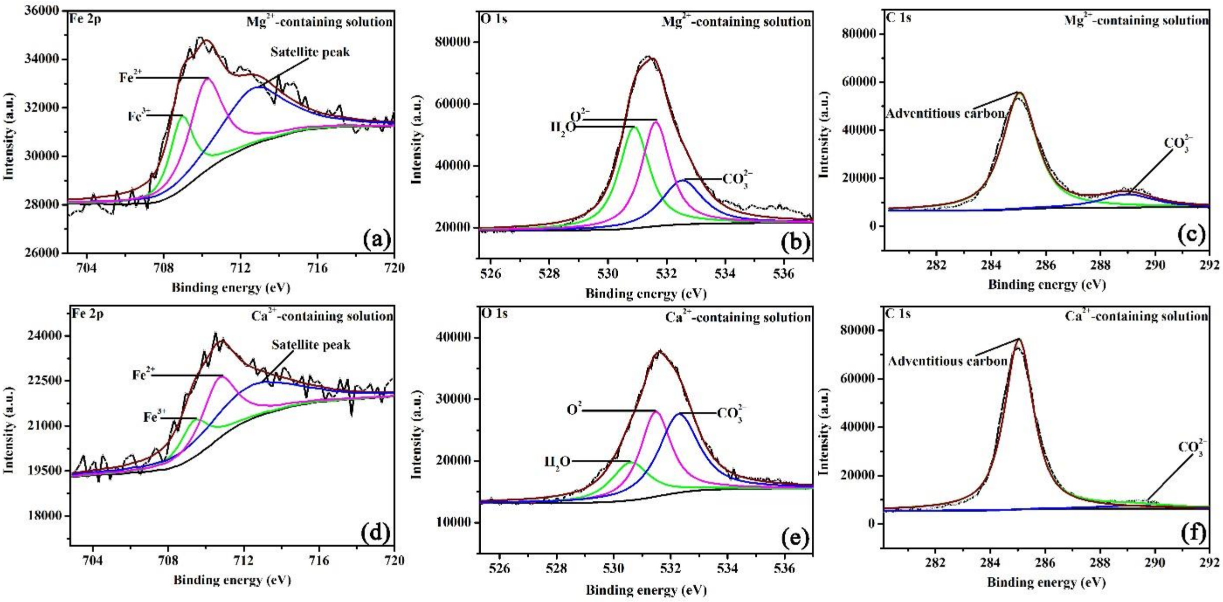

Figure S2. The XPS pattern of the corrosion scales of the specimens exposed to the blank solutions with Mg^{2+} addition (a, b, and c) and Ca^{2+} addition (d, e, and f) for 48 h.

1.3. Effects of Mg^{2+} and Ca^{2+} on the longer-term corrosion behavior of X100

The phase angle and Bode representation of Fig. 9 in the main text are given in Fig. S3. The presence of Ca^{2+} raises the values of impedance moduli $|Z|$, whereas the Mg^{2+} in solution decreases the values of $|Z|$. In the literature, an equivalent circuit of *R(RQ)* was utilized to fit the EIS spectra for CO_2 corrosion scale of steel [1]. *Q* corresponds to a constant phase element (CPE), and the interfacial behaviors of non-ideal capacitive are commonly interpreted by CPE [2]. Only the electric double layer behavior is considered by this model. The scale formation which can significantly influence the impedance response was not considered by this model. Furthermore, EIS spectra of X100 placed in the CO_2 saturated solution was simulated by *R(Q(R(C(R))))* equivalent circuit, as indicated by Sun *et al.* [3]. However, the diffusion behavior at low frequency was not considered. Consequently, $R_s(Q_{dl}(R_{ct}(Q_{cs}(R_{cs}W))))$ was employed to explain the solution/steel interface of this study [4]. The fitting results are all provided in Table S.1. The values of Chi-Square (X^2) are approximate 1×10^{-4}, demonstrating that the measured date match with the chosen equivalent circuit.

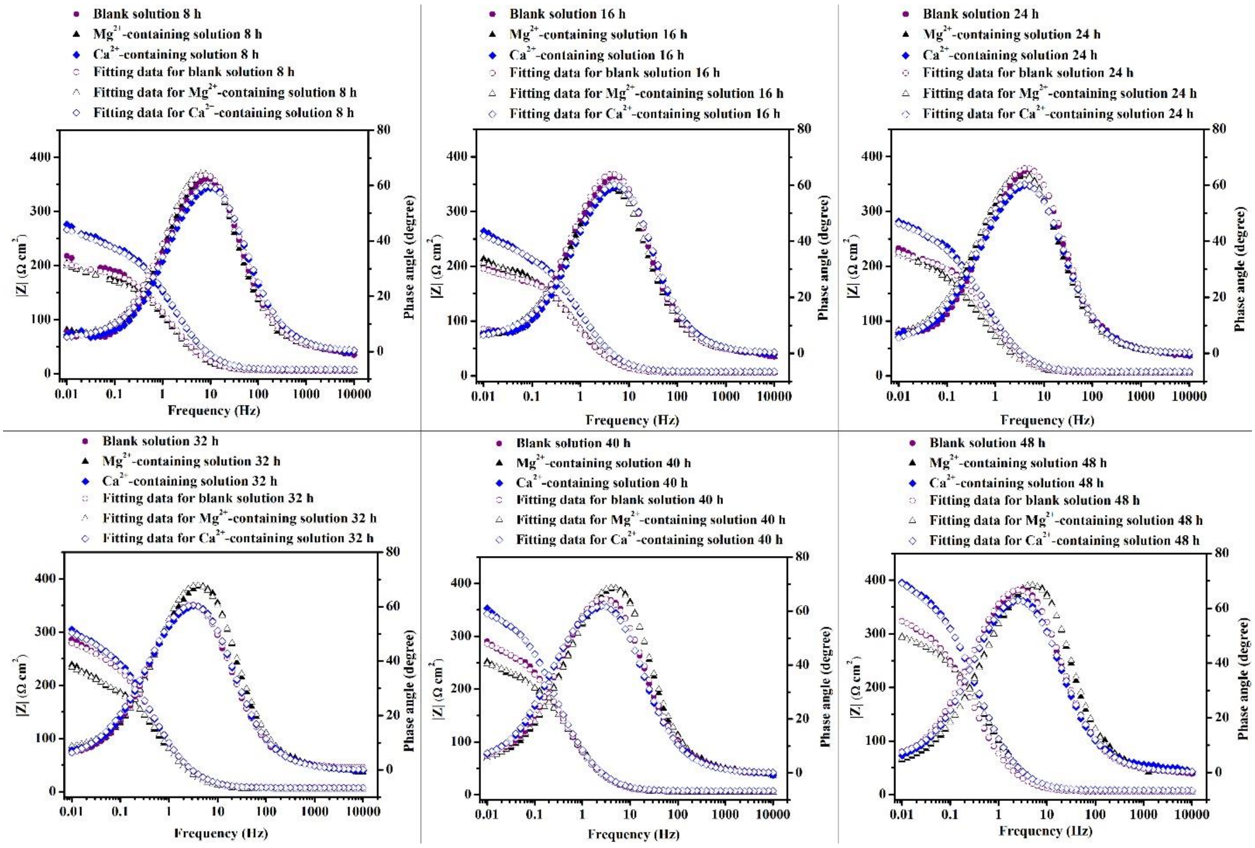

Figure S3. Bode plots for the specimens placed in the blank solutions with various additions.

Table S1. The fitting results of EIS spectra for the specimens in the blank solutions with various additions. χ^2 is the Chi-square.

Components	R_s Ω cm^2	Q_{dl}×10^3 F cm^{-2}	n	R_{ct} Ω cm^2	Q_{cs}×10^3 F cm^{-2}	n	R_{cs} Ω cm^2	W×10^{-4} $Ω^{-1}s^{0.5}$	χ^2×10^{-4}
Blank solution 8h	5.802±1.105	0.851±0.014	0.965±0.360	127.8±4.4	5.13±1.74	0.8557±0.173	52.09±10.43	1502±104	2.207±0.047
Blank solution 16h	5.622±0.067	1.461±0.249	0.969±0.016	124±10.7	5.405±0.464	0.97±0.283	40.81±4.63	1125±48	7.373±0.174
Blank solution 24h	5.442±0.356	1.431±0.446	0.973±0.179	140.8±11.7	4.279±0.004	0.914±0.291	567.1±19.6	1171±34	5.979±0.085
Blank solution 32h	2.928±0.009	1.531±0.539	0.71±0.048	5.353±0.192	3.608±0.564	0.922±0.103	35.57±6.73	3171±131	1.819±0.381
Blank solution 40h	5.985±0.589	1.943±1.045	0.932±0.284	40.86±0.16	3.199±0.289	0.5218±0.016	259.5±60.4	12.5±1.2	3.418±0.0895
Blank solution 48h	5.36±2.245	2.042±0.746	0.942±0.038	0.01±0.00	2.911±0.035	0.6947±0.153	277.9±43.5	12.6±1.4	2.460±0.104
Mg^{2+}-containing solution 8h	0.857±0.065	5.961±0.493	0.609±0.325	5.297±0.372	8.48±3.08	0.9709±0.174	78.4±10.3	8.24±0.32	14.93±0.694
Mg^{2+}-containing solution 16h	6.816±3.432	5.612±0.794	0.78±0.004	1.01±0.35	7.545±0.964	0.9378±0.382	478.2±74.5	4.31±0.48	9.007±0.048
Mg^{2+}-containing solution 24h	5.279±0.026	1.937±0.034	0.954±0.165	76.83±0.58	7.16±1.94	0.3901±0.274	40.08±1.1	35.63±0.15	6.420±0.784

Mg^{2+}-containing solution 32h	4.589±0.019	1.671±0.493	0.975±0.194	1.07±0.26	5.460±2.642	0.8272±0.463	104±4.5	1.104±0.05	5.688±0.595
Mg^{2+}-containing solution 40h	4.546±0.854	1.436±0.377	0.832±0.174	87.72±10.47	5.64±1.64	0.813±0.184	80.7±7.1	41.11±1.54	4.354±.0369
Mg^{2+}-containing solution 48h	4.296±1.104	3.197±0.489	0.81±0.438	7.506±0.473	3.022±1.455	0.92±0.041	107.8±10.6	14.17±0.07	3.218±0.529
Ca^{2+}-containing solution 8h	8.071±0.836	0.631±0.019	0.923±0.384	40.36±0.15	5.261±2.743	0.7548±0.173	366.5±5.1	2.86±0.42	9.392±0.007
Ca^{2+}-containing solution 16h	2.312±0.697	4.464±0.026	0.726±0.138	5.756±1.307	5.182±0.643	0.9375±0.143	364±4.7	3.48±0.95	5.124±0.058
Ca^{2+}-containing solution 24h	7.742±1.286	1.287±0.094	0.937±0.345	0.01±0.00	3.675±0.154	0.8311±0.302	184±40.2	0.103±0.024	4.587±0.216
Ca^{2+}-containing solution 32h	7.731±1.180	1.592±0.694	0.925±0.473	0.01±0.00	2.76±0.15	0.72125±0.103	214.5±4.1	9.74±0.04	2.710±0.049
Ca^{2+}-containing solution 40h	7.499±1.543	1.93±0.483	0.912±0.046	198.4±0.4	2.217±0.368	0.7894±0.053	128.7±20.5	1541±105	1.386±0.084
Ca^{2+}-containing solution 48h	7.22±1.574	2.875±0.603	0.702±0.139	8.516±3.684	1.60±0.74	0.8945±0.083	0.08124±33.6	2320±75	5.191±0.316

Fig. S4, 5, and 6 show the cross-section morphologies of the scales in the three solutions. The corrosion of substrate of X100 pipeline steel is not homogeneous, owing to the preferential dissolution of ferrite [5]. In order to obtain reliable average thickness under each condition, at least ten different positions in each figure were examined and the average thicknesses were calculated (given in Fig. 12 of the main text). Here, for simplicity, only the minimum and maximum thicknesses in each figure were marked (Fig. S4, 5, and 6). The scales on the specimens in solutions containing Mg^{2+} are thinner than those formed in the blank solutions. When the Ca^{2+} is added to solutions, the average thicknesses of corrosion scales are increased.

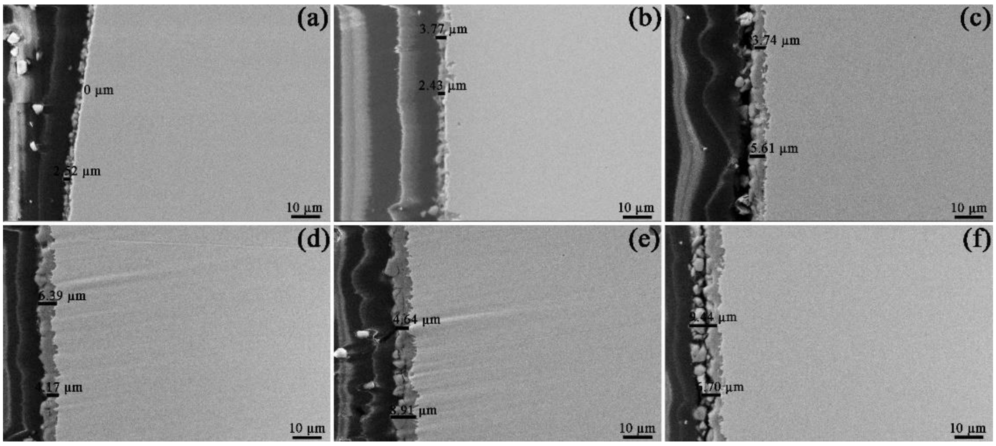

Figure S4. The cross-section morphologies of the scales formed in the blank solutions: (a) 8 h, (b) 16 h, (c) 24 h, (d) 32 h, (e) 40 h, and (f) 48 h.

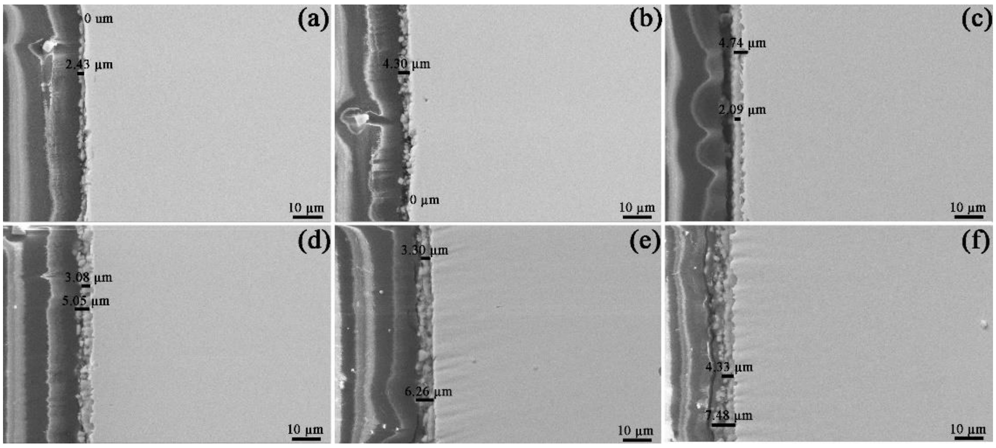

Figure S5. The cross-section morphologies of the scales formed in Mg^{2+}-containing solutions: (a) 8 h, (b) 16 h, (c) 24 h, (d) 32 h, (e) 40 h, and (f) 48 h.

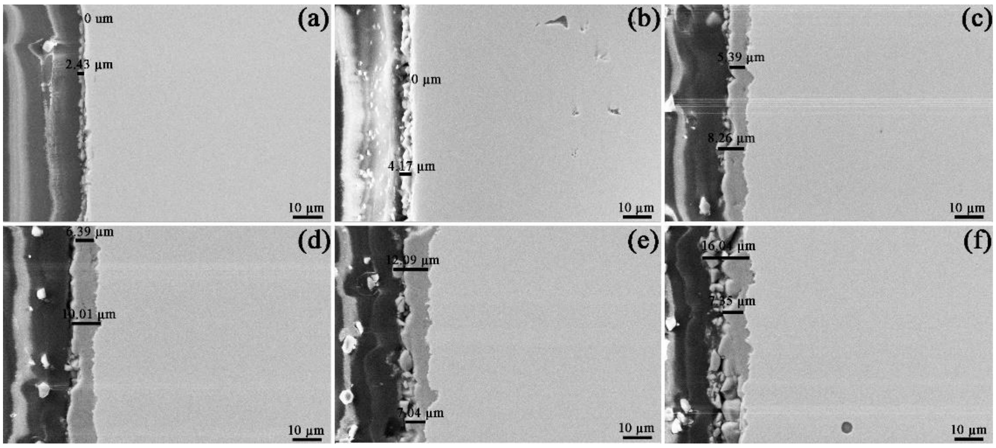

Figure S6. The cross-section morphologies of the scales formed in Ca^{2+}-containing solutions: (a) 8 h, (b) 16 h, (c) 24 h, (d) 32 h, (e) 40 h, and (f) 48 h.

References

1. D.A. López, S.N. Simison, S.R. de Sánchez, The influence of steel microstructure on CO_2 corrosion. EIS studies on the inhibition efficiency of benzimidazole, Electrochim. Acta, 48 (2003) 845–854.
2. J. Liu, A. Alfantazi, E. Asselin, A new method to improve the corrosion resistance of titanium for hydrometallurgical applications, Appl. Surf. Sci., 332 (2015) 480–487.
3. J.B. Sun, G.A. Zhang, W. Liu, M.X. Lu, The formation mechanism of corrosion scale and electrochemical characteristic of low alloy steel in carbon dioxide-saturated solution, Corros. Sci., 57 (2012) 131–138.
4. I.M. Gadala, A. Alfantazi, A study of X100 pipeline steel passivation in mildly alkaline bicarbonate solutions using electrochemical impedance spectroscopy under potentiodynamic conditions and Mott–Schottky, Appl. Surf. Sci., 357 (2015) 356–368.
5. F.F. Eliyan, A. Alfantazi, On the theory of CO_2 corrosion reactions–Investigating their interrelation with the corrosion products and API-X100 steel microstructure, Corros. Sci., 85 (2014) 380–393.
